# The Brazilian biofuels industry

**DOI:** 10.1186/1754-6834-1-6

**Published:** 2008-05-01

**Authors:** José Goldemberg

**Affiliations:** 1 University of São Paulo, Institute of Electrotechnics and Energy, Av. Prof. Luciano Gualberto, São Paulo, SP 05508-010, Brazil

## Abstract

Ethanol is a biofuel that is used as a replacement for approximately 3% of the fossil-based gasoline consumed in the world today. Most of this biofuel is produced from sugarcane in Brazil and corn in the United States. We present here the rationale for the ethanol program in Brazil, its present 'status' and its perspectives. The environmental benefits of the program, particularly the contribution of ethanol to reducing the emission of greenhouse gases, are discussed, as well as the limitations to its expansion.

## Introduction

Fuel-grade ethanol, produced from biomass, has been considered as a suitable automotive fuel for nearly a century, particularly for vehicles equipped with spark-ignition engines (technically referred to as Otto cycle engines, but commonly known as gasoline engines). Ethanol was not used in significant amounts until the mid 1970s. The dramatic increase in the cost of oil at the time of the first oil crisis imposed severe foreign exchange burdens on countries dependent upon oil imports, including Brazil. As a leading producer of sugar from sugarcane, Brazil was well situated to explore the option of ethanol as an alternative to gasoline. This led the Government to encourage the redirection of some sugarcane production to generate ethanol as a replacement for gasoline, thus reducing oil imports.

Under the Brazilian Government's plan, PETROBRAS, the state-owned oil company, would purchase a guaranteed amount of ethanol from producers. In addition, economic incentives were given to agro-industrial enterprises willing to produce ethanol, in the form of low interest rates. This translated into nearly US$2.0 billion in loans from 1980 to 1985, representing 29% of the total investment needed [1]. On the basis of such policies, ethanol production increased rapidly over the years, reaching 18 billion liters in 2007.

Ethanol from sugarcane, produced under proper conditions, is essentially a renewable fuel and has clear advantages over gasoline in reducing greenhouse gas emissions and improving air quality in metropolitan areas.

In this paper we review the technological characteristics of ethanol as a fuel, the present 'status' of the ethanol Program in Brazil, the characteristics of ethanol as a renewable fuel, the future perspectives of the ethanol Program in Brazil followed by a discussion on the possibility of expanding ethanol production from sugarcane and conclusions.

### Technical characteristics of ethanol as a fuel

Ethanol is an excellent motor fuel. It has a motor octane number of 98 which exceeds that of gasoline (octane number of 80). It also has a lower vapor pressure than gasoline, which results in lower evaporative emissions. Ethanol's flammability in air is also lower than that of gasoline which reduces the number and severity of vehicle fires. Anhydrous ethanol has lower and higher heating values of 21.2 and 23.4 MJ/liter, respectively; for gasoline the values are 30.1 and 34.9 MJ/liter (see [2]).

On the basis of higher heating value, ethanol has only 67% of the energy content compared with the same volume of gasoline. However, since it has a motor octane number higher than gasoline, it can be used in engines with a higher compression ratio (12-to-1, compared with the 8-to-1 ratio typically found in gasoline-fueled engines). As a result, ethanol-fueled engines are approximately 15% more efficient than motors using gasoline, which compensates to some extent for the lower energy content per unit volume [3]. Typically, one would require approximately 20% more ethanol than gasoline per kilometer driven.

In Brazil ethanol was used initially in one of two ways:

• blended as an octane enhancer in gasoline; typical blends range from 20% to 25% anhydrous (a mixture called gasohol; anhydrous ethanol is 99.6% ethanol Gay-Lussac (GL) which express the percentage of alcohol in a blend) and 0.4% water; ethanol in fuel by volume, or

• on its own, in neat-ethanol engines; used in the form of hydrous ethanol at 95.5 GL.

Ethanol's properties (as a fuel) have led to the development of dedicated engines for neat ethanol in Brazil. Initial efforts were conducted by the Centro de Tecnologia Aeronáutica (CTA) in São Paulo state, where most of the tests with engines running with ethanol-gasoline blend and straight ethanol were performed up to 1980 [3].

In the early 1980s, vehicles with neat-ethanol engines that could use hydrous ethanol became highly attractive to consumers, as the government had ensured the pump price of hydrous alcohol would be equivalent to 64.5% of the gasoline price. Sales of neat-ethanol powered vehicles exploded, and the market share occupied by these vehicles increased to more than 90% of all vehicle sales. The total fleet of neat-ethanol fueled vehicles at one point reached 5 million. Most of the service stations were equipped with two reservoirs (one for anhydrous ethanol, and the other for ethanol-gasoline blends at 20% to 25% ethanol). The lack of a guarantee of ethanol production to supply this expanding market became a critical issue, and in the early 1990s a shortage in ethanol production led to a serious crisis and a gradual abandonment of the use of neat-ethanol driven cars. There were other problems associated with neat-ethanol vehicles, including the problem of not being able to use them in neighboring countries or in regions of Brazil which did not have service stations capable of supplying pure ethanol.

The introduction of flex-fuel motors in Brazil, in 2003, solved this problem, since they are capable of running with blends from E0 to E100. The technology is based on sensors in the fuel system that automatically recognizes the ethanol level in the fuel. The engine's electronic control unit then self-calibrates for the best possible operation; if ethanol is not present, the engine will self-calibrate to gasoline-only operation. The process is instantaneous and undetectable by the vehicle driver.

### Present 'status' of the ethanol program in Brazil

Presently there are 325 plants in operation crushing 425 million tons of sugarcane per year, approximately one-half being used for sugar and the other half for ethanol production. Approximately 17.8 billion liters of ethanol were produced in 2006, using 2.9 million hectares of land (Table 1).

**Table 1:** Ethanol distilleries in Brazil [15] (147 new distilleries are in the planning stages, 86 of which due to be completed up to 2015).

	**Distilleries (units)**	**Cane crushed (millions of tons)**	**Average size (million tons)**
Brazil (total)	325	425	1.3
São Paulo	148	264 (62% of total)	1.8
Northeast	74	53	0.7

A typical plant crushes 2 million tons of sugarcane per year and produces 200 million liters of ethanol per year (1 million liters per day over 6 months, April to November) and costs approximately US$150 million. The planted area required to supply the sugarcane is typically 30,000 hectares.

**Figure 1:**
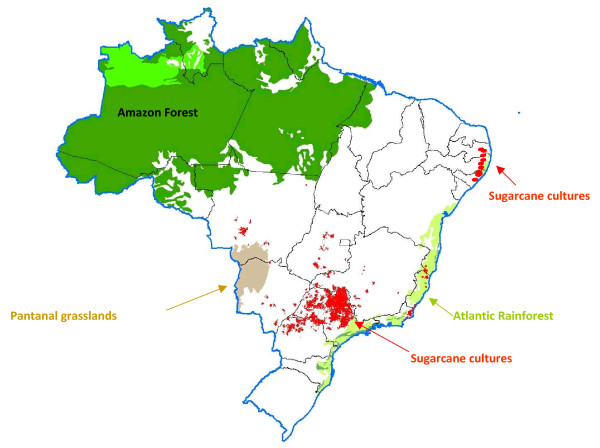
Map of sugarcane crops in Brazil.

Most of the large plants are located in the state of São Paulo, where almost two-thirds of the ethanol is being produced. This state is located at a distance from the Amazonia region, as shown in Figure 1.

**Figure 2:**
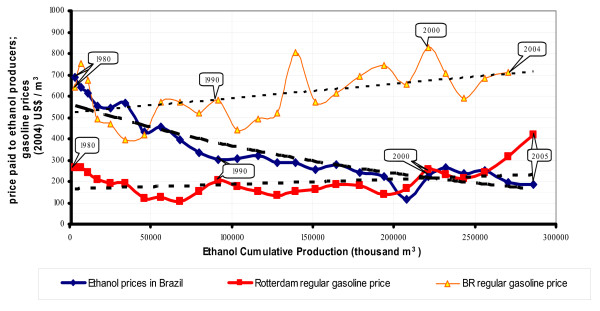
The economic competitiveness of alcohol fuel compared with gasoline [21].

Ethanol production in Brazil was initiated with a highly subsidized program. The price paid to producers in 1980 was US$700 for 1000 liters; over the intervening years, gains in technology and economies of scale have driven the cost down, reaching as low as US$200 per 1000 liters in 2004 (Figure 2).

By 2004, ethanol in Brazil had become economically competitive with gasoline based on international prices for oil (equivalent to US$40 per barrel). At these costs, the production of ethanol from sugarcane is much cheaper that from other crops such as corn, wheat and sugarbeet (Table 2)

**Table 2:** Comparison of the production costs (€/1000 liters) of ethanol in Brazil, United States and Germany [19].

	**USA Corn**	**Germany Wheat**	**Germany Sugarbeet**	**Brazil Sugarcane**
Buildings	00.39	00.82	00.82	00.21
Equipment	03.40	05.30	05.30	01.15
Labor	02.83	01.40	01.40	00.52
Insurance, taxes and other costs	00.61	01.02	01.02	00.48
Feedstock	20.93	27.75	035.10	09.80
Other operation costs	11.31	18.68	015.93	02.32

**Total production cost**	**39.47**	**54.97**	**59.57**	**14.48**

Sale of byproducts	06.71	06.80	07.20	
Government subsidies	07.93			

**Net production cost**	**24.83**	**48.17**	**52.37**	**14.48**

### Characteristics of ethanol as a renewable fuel

#### The impact of alcohol engines on air pollution

Presently all gasoline used in Brazil is blended with 25% anhydrous ethanol, a fuel with lower toxicity than fossil fuels [3].

Lead additives to gasoline were reduced through the 1980s as the amount of ethanol blended in the fuel was increased, and these additives were completely eliminated by 1991. The additions of aromatic hydrocarbons (such as benzene), which are particularly toxic, were also eliminated, and the sulfur content was reduced (in vehicles using blended fuel) or eliminated (in neat-ethanol fueled vehicles) [3]. The addition of ethanol instead of lead to gasoline has lowered the total carbon monoxide (CO), hydrocarbons and sulfur emissions significantly. Exhaust emissions associated with ethanol are less toxic than those associated with gasoline, and have lower atmospheric reactivity [4]. The use of ethanol has also reduced CO emissions drastically. Before the Brazilian Alcohol Program started, when gasoline was the only fuel in use, CO emissions were higher than 50 g/km driven; they had been reduced to less than 5.8 g/km in 1995. Lead ambient concentrations in São Paulo Metropolitan Region dropped from 1.4 μg/m^3 ^in 1978 to less than 0.10 μg/m^3 ^in 1991, according to CETESB (the Environmental Company of São Paulo State), which is far below the air quality standard of 1.5 μg/m^3 ^(see [5]).

One of the drawbacks of the use of hydrous ethanol in neat-ethanol engines is the increase in aldehyde emissions as compared with gasoline or gasohol. It can be argued, however, that the acetaldehyde from alcohol use is less aggressive to human health and the environment than formaldehyde produced when gasoline is used [5]. Total aldehyde emissions from engines using ethanol (both neat and blended) are higher than those using gasoline, but it should be noted that these are predominantly acetaldehydes. The present ambient concentrations of aldehyde, in São Paulo, are below the reference levels recommended as adequate to human health found in the literature [6].

#### The net energy balance

The net energy balance for ethanol is defined here as the ratio of the energy contained in a given volume of ethanol divided by the fossil energy required for its production (in the form of fertilizers, pesticides, diesel fuel spent in mechanized harvesting and the transportation of sugarcane to the processing mill). Sugarcane is made up of three components: sucrose, bagasse, and tops and leaves.

**Figure 3:**
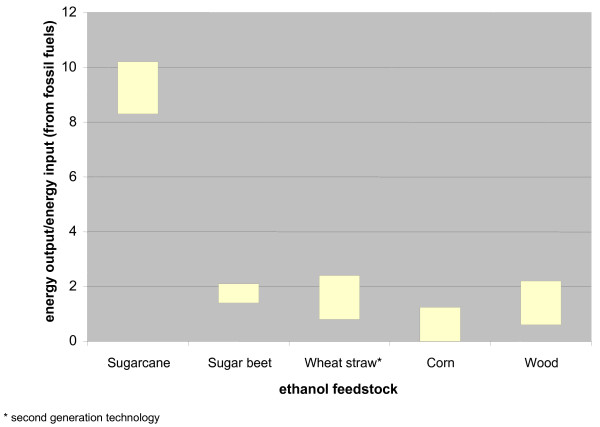
Energy balance of alcohol production from different feedstocks [8, 9, 22].

Bagasse contains one-third of the energy in the sugarcane, and is the source of all of the energy needed in the ethanol mills. The other two-thirds are split between sucrose and the tops and leaves [7]. As a consequence the net energy balance for ethanol production is high, between 8.2 and 10, much better than the net energy balance in the production of ethanol from corn, which requires significant fossil fuel inputs and stands at approximately 1.3 (Figure 3) [8,9]. (The data for the energy balance from wheat straw using second-generation technology is based on laboratory and pilot plant projects and is, therefore, controversial.)

The positive energy balance associated with sugarcane-based ethanol is reflected by a considerable reduction (91%) in greenhouse gas emissions resulting from replacing gasoline with this biofuel. By comparison, ethanol from corn-based production is estimated to provide an 18% reduction in greenhouse gas emissions [10]. The use of ethanol as a replacement for gasoline has led to an overall reduction of 9.2 million tons of carbon per year in carbon emissions in Brazil (10% of the total) [11,12].

### Future perspectives of the ethanol program in Brazil

Presently the production of ethanol in Brazil relies exclusively on first-generation technologies that are based on the utilization of the sucrose content of sugarcane. As discussed above, sucrose represents only one-third of the energy content of sugarcane.

The efficiency of sugarcane-to-ethanol production can still be increased through improvements in the agricultural and industrial phases of the production process. For example, in the agricultural phase, good sugar cane yield and a high index of TRS (total recoverable sugar) are the main drivers for high yield of ethanol per unit of planted area. The increase of TRS from sugarcane has been very significant: 1.5% per year in the period 1977–2004, resulting in an increase from 95 to 140 kg/ha. Sugar extraction from sugar cane has also increased in the period 1977–2003. The average annual improvement was 0.3%; some mills have already reached extraction efficiencies of 98% (see [4,13]).

**Figure 4:**
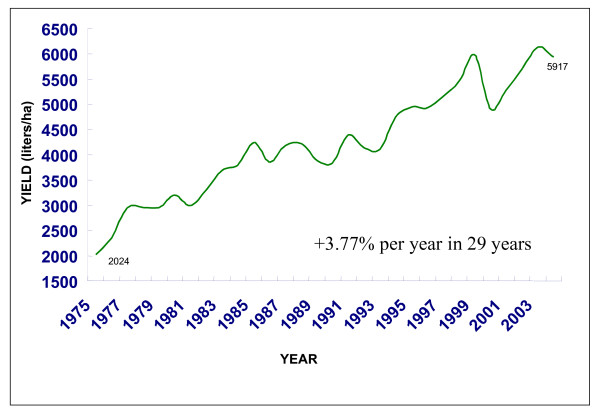
Ethanol productivity in Brazil.

Figure 4 shows the evolution of the ethanol yield between 1975 and 2004; in 29 years, the efficiency of ethanol production has grown by 3.77% per year.

In the State of São Paulo, an increase of 12% in sugar cane yield and 6.4% in TRS is expected over the next 10 years. Combining this with an expected 6.2% improvement in fermentation efficiency and 2% in sugar extraction, ethanol yields may increase by 29%, pushing average ethanol productivity to 9000 litres/ha (see [4,14]).

Another important perspective of the present ethanol program in Brazil is the production of large amounts of electricity from the burning of bagasse with improved technologies. In the early days of the program, bagasse was burned inefficiently to produce the heat needed for the industrial part of the process (crushing fermentation and distillation) and there was a huge excess of waste bagasse. Today, high-pressure boilers operate at close to 100 bar (in contrast to the low-pressure, 20-bar units used in the past); as a result, energy recovery has increased and there is in fact a large electricity surplus that is supplied to the grid [15]. Table 3 gives an indication of the expected electricity surplus that will be supplied to the grid in 2015.

**Table 3:** Cogeneration potential (burning sugarcane bagasse and trash in 65-bar boilers)

	Installed capacity (MW)	Surplus to the grid (MW)
Season 2015/2016	15,750	11,018

## Discussion

As mentioned in previously, there are 2.9 million hectares of sugarcane in use for the production of ethanol in Brazil; another 3.2 million hectares are used for sugar production. Table 4 shows land use in Brazil indicating that sugarcane uses less than 10% of agricultural area.

**Table 4:** Land use in Brazil [20]

	Area (million hectares)	Percentage
Soy	21	7
Corn	12	4
Sugarcane	5.4	2
Other cultures	17	6
Total agriculture	60	20
Pastureland	237	80
Agriculture + pastureland	297	100

The predicted increased use of flex-fuel cars would suggest that demand for ethanol will double in approximately 10 years in Brazil. The private sector is planning to build the plants needed for increased production, which will double the land area used for sugarcane plantations (mainly in the State of São Paulo). This will be done by converting existing pastureland, of which there are 237 million hectares [16,17].

There are concerns that such expansion could generate an indirect pressure pushing cattle into the Amazonia leading to further deforestation in that area, but there is no direct evidence for that. On the contrary, what has happened is that the density of cattle on pastureland has increased from 1.28 heads of cattle/hectare in 2001 to 1.41 heads of cattle/hectare in 2005 [18].

On a worldwide scale, other sugar-producing countries such as India, China, Thailand, Pakistan, Mexico, Columbia and South Africa could produce significant amounts of ethanol. Colombia already has four large distilleries in operation.

## Conclusion

The Ethanol Program in Brazil is firmly established today, and is replacing approximately 40% of the gasoline that would be otherwise be consumed in the country, at a competitive prices, using 2.9 million hectares of land. This has led to improvements in the air quality of the São Paulo metropolitan area, and reductions in greenhouse gas emissions.

The ethanol program in Brazil has replaced approximately 1.5% of all gasoline used in the world, and this figure will most likely double with the expansion underway. If the present rate of growth of ethanol production in Brazil continues and if other sugar-producing countries follow the route adopted by Brazil, it seems possible that as much as 10% of all gasoline used in the world could be replaced in the next 15–20 years.
